# The Role of Single-Layered Flap in Temporal Inverted Internal Limiting Membrane Flap Technique for Macular Holes: Pros and Cons

**DOI:** 10.1155/2019/5737083

**Published:** 2019-06-02

**Authors:** Yasuyuki Takai, Masaki Tanito, Kazunobu Sugihara, Akihiro Ohira

**Affiliations:** ^1^Department of Ophthalmology, Shimane University Faculty of Medicine, Izumo 693-8501, Japan; ^2^Division of Ophthalmology, Masuda Red Cross Hospital, Masuda 698-8501, Japan

## Abstract

**Purpose:**

To assess the safety and effectiveness of the single-layered inverted internal limiting membrane (ILM) flap technique for treating chronic, large, or highly myopic macular holes (MHs).

**Methods:**

The medical records of 20 eyes of 20 consecutive Japanese patients with large MHs (*n*=6) (minimal diameter, >400 *μ*m), chronic MHs (*n*=2) (symptom duration, >24 months), MHs in high myopia (*n*=11) (axial length, >26 mm), and MHs in a patient unable to maintain prone positioning postoperatively (*n*=1) were reviewed retrospectively. All patients underwent 25-gauge pars plana vitrectomy and the temporal inverted ILM flap technique. A semicircular ILM notch was made temporally two disc diameters from the MH using a 25-gauge knife, and the ILM was peeled temporally to create a semicircular ILM flap using a 25-gauge forceps. The single-layered ILM flap was inverted in a nasal direction to cover the MH. When an epiretinal membrane (ERM) was present, it was peeled before the ILM flap was inverted.

**Results:**

The MHs closed successfully in all (100%) eyes postoperatively. In the MHs associated with an ERM, after hole closure, gradual foveal deformation occurred in both the area from which the ILM was not peeled and the ILM flap inverted side.

**Conclusions:**

The single-layered inverted ILM flap technique, a simple surgery to treat MHs, provides scaffolding for retinal gliosis and may facilitate bridge formation between the walls of the MH under the flap. Considering the 100% success rate of MH closure, this technique seems to be effective and safe for treating chronic, large, or highly myopic MHs and MHs in patients unable to maintain postoperative prone positioning. In the MHs associated with ERMs, gradual foveal deformation was observed after ERM peeling. Further studies are needed to minimize surgical complications and understand the mechanism of this technique. This trial is registered with UMIN000035091.

## 1. Introduction

Internal limiting membrane (ILM) peeling has become a standard procedure in the surgical treatment of idiopathic macular holes (MHs) in recent years. The satisfactory results with single-procedure MH closure rates of 50% to 88% for large MHs exceeding 400 *μ*m [1, 2], 40% to 83% for chronic MHs [3, 4], 63% to 90% for MHs with high myopia [5–7], and 60% to 100% without postoperative face-down positioning [8, 9] have been reported; however, surgical closure of these MHs remains challenging. Michalewska et al. first reported the inverted ILM flap technique as a new surgical strategy that was performed successfully to treat large MHs and MHs in highly myopic eyes for which a poor single-procedure MH closure rate was expected [10, 11]. The temporal inverted ILM flap technique, a modified form in which the ILM is peeled from the temporal side of the fovea only was performed to treat large MHs. This procedure recently was reported to decrease the risk of surgical trauma and dissociated optic nerve fiber layer appearance with the same anatomic and functional results compared with the original inverted ILM flap technique [12]. We reported a case of a successfully performed temporal inverted ILM flap technique combined with intraocular sulfur hexafluoride (SF_6_) gas tamponade to treat a patient with a MH who could not maintain postoperative prone positioning [13]. Recently, various modifications of the original inverted ILM flap technique have been reported, e.g., covering or filling the MH with the ILM flap and inverting from all sides or less. Because the same anatomic and functional results have been achieved compared with the ILM peeling technique [14], the indications for the inverted ILM flap technique have been widening. However, some complications related to the inverted ILM flap technique have been reported, including worse postoperative visual acuity (VA) than the preoperative level [15, 16], expansion of retinal pigment epithelial (RPE) atrophy [17], and prevention of MH closure and/or functional recovery of the outer retina by the inverted ILM flap [18]. The current study reports the effectiveness of the single-layered inverted ILM flap technique performed to treat chronic, large, or highly myopic MHs and MHs in patients unable to maintain postoperative prone positioning and foveal deformation that have not been reported previously.

## 2. Methods

The current study was part of the study protocol “Epidemiologic Study of Ocular Morphology and Function,” that the Ethics Committee of Shimane University Hospital approved. The study adhered to the tenets of the Declaration of Helsinki. The ethics committee waived the requirement for informed consent regarding the use of patients' medical record data in accordance with the regulations of the Japanese Guidelines for Epidemiologic Study issued by the Japanese Government, and instead, the protocol was posted at the outpatient clinic to inform participants of the study.

We retrospectively reviewed the medical records of 20 consecutive Japanese patients with large MHs (minimal diameter, >400 *μ*m), chronic MHs (symptomatic duration, >24 months), MHs in high myopia (axial length, >26 mm), and MHs in patients who could not maintain postoperative prone positioning after vitrectomy. These patients underwent the temporal inverted ILM flap technique with gas tamponade with SF_6_ at Shimane University Hospital from March 2014 to February 2018. All patients underwent thorough ophthalmologic examinations that included measurement of the best-corrected VA (BCVA) using a Landolt decimal acuity chart, the results of which were converted to the logarithm of the minimal angle of resolution VA; axial length measured by AL-3000 ultrasound biometry (Tomey, Nagoya, Japan); indirect ophthalmoscopy; and three-dimensional optical coherence tomography (OCT)-2000 spectral-domain OCT (Topcon, Tokyo, Japan). The three-dimensional scan had a resolution of 512 × 128 and a scan length of 6.0 × 6.0 mm. The MH measurements included determination of the maximal diameter, defined as the longest distance of the MH base, and the minimal diameter, defined as the shortest distance between the MH edges. Patients with a history of retinal surgery, retinal detachment, diabetic retinopathy, retinal vascular occlusion, uveitis, and trauma were excluded.

### 2.1. Surgical Method

**Figure 1:**
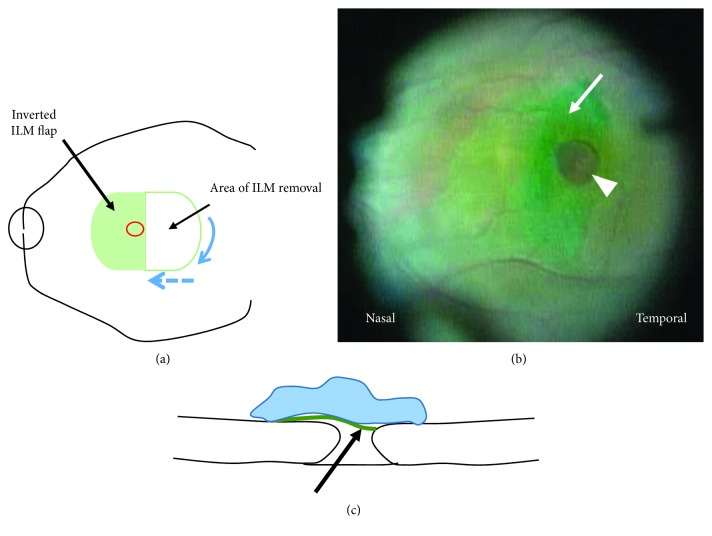
Schematic drawing of the temporal ILM flap technique. After a semicircular ILM notch (blue arrow) is created two disc diameters from the temporal edge of MH (red circle), the ILM is peeled and inverted in the nasal direction to fully cover the MH (blue dashed arrow) (a). An indocyanine green-stained temporal ILM flap created at the temporal macular is inverted in the nasal direction (arrow) to cover the MH (arrowhead) (b). A cross-sectional drawing of the temporal ILM flap technique. The temporal ILM flap (arrow) is inverted toward the nasal retina to cover the MH and is stabilized with a low-molecular-weight viscoelastic material (arrowhead) (c).  Figure 1(a) is reproduced from Takai et al. [13] (under the Creative Commons Attribution License/public domain).

A 25-gauge pars plana vitrectomy combined with the temporal inverted ILM flap technique which have previously been described [13] was performed. Core vitrectomy and ILM staining with a 0.125% solution of indocyanine green were performed. If an epiretinal membrane (ERM) was present, it was peeled. Then, a 25-gauge MVR knife (MANI, Utsunomiya, Japan) was used to create a semicircular ILM notch two disc diameters from the MH in a temporal area, and the 25-gauge end grasping forceps (Alcon, Fort Worth, TX, USA) was used to peel ILM in the temporal macula to create a semicircular ILM flap and to invert the single-layered ILM flap in a nasal direction to fully cover the MH (Figures 1(a) and 1(b)). Next, a low-molecular-weight viscoelastic material (Viscoat, Alcon) was placed on the inverted flap to stabilize it (Figure 1(c)). After fluid-air exchange, 20% SF_6_ gas was injected into the vitreous cavity. Phacoemulsification with intraocular lens implantation was performed simultaneously in all phakic patients because of the development of cataracts that hinder visual improvement after vitrectomy. The patients, except for a patient who could not maintain postoperative prone positioning, were advised to maintain a face-down position for 2 days postoperatively. Y. T. performed all surgeries. The surgical techniques are shown in Supplementary video (available here).

### 2.2. Statistical Analysis

Statistical analyses were performed using JMP version 11 software (JMP Statistical Discovery, Cary, NC, USA). The difference between the preoperative and postoperative BCVAs was compared using the Wilcoxon signed-rank test.

## 3. Results

Twenty eyes of 20 patients (12 women, 8 men; mean age, 66.8 ± 9.9 years; range, 48–82 years) were included in this study. Six patients had large MHs, two patients had chronic MHs, 11 patients had MHs with high myopia, and one patient could not maintain postoperative prone positioning. The characteristics of the 20 eyes are summarized in Table 1.

ERMs were present with the MHs in four (20%) eyes. Phacoemulsification with intraocular lens implantation was performed in all 14 phakic eyes. The postoperative follow-up time ranged from 6 to 36 months.

**Figure 2:**
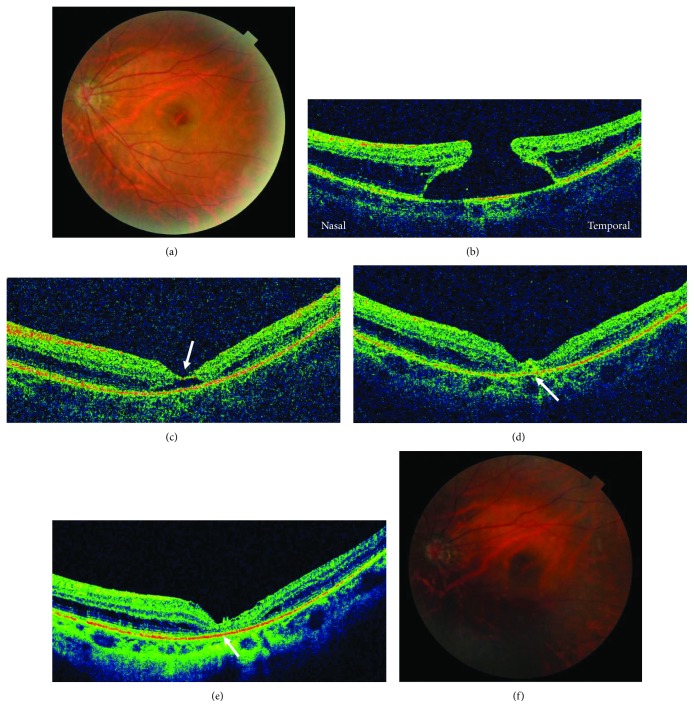
Preoperative and postoperative fundus and OCT images. A 55-year-old woman with a MH in high myopia (a). The preoperative minimal and maximal MH diameters are 604 *μ*m and 2,296 *μ*m, respectively (b). The axial length is 27.8 mm and the decimal BCVA is 0.04. At postoperative week 2, the MH remains opens under the covered ILM flap (flap closure) (arrow) (c). At postoperative week 4, the MH edges form a bridge under the ILM flap (arrow) (d). At postoperative week 6, the MH is closed leaving a partially defective ELM and EZ (arrow) (e, f). The decimal BCVA is 0.1.

Regarding anatomic outcomes, the MH closure rate was 100% after the first surgery (Table 2). In two cases with chronic MHs, two cases with MHs in high myopia, and one case of a patient with a MH who could not maintain postoperative prone positioning, MH closure as a result of only the single-layered inverted ILM flap, referred to as “flap closure,” was observed at two weeks after surgery (Figure 2(c)). A representative case of a 55-year-old woman with a MH in high myopia is shown in Figure 2.

**Figure 3:**
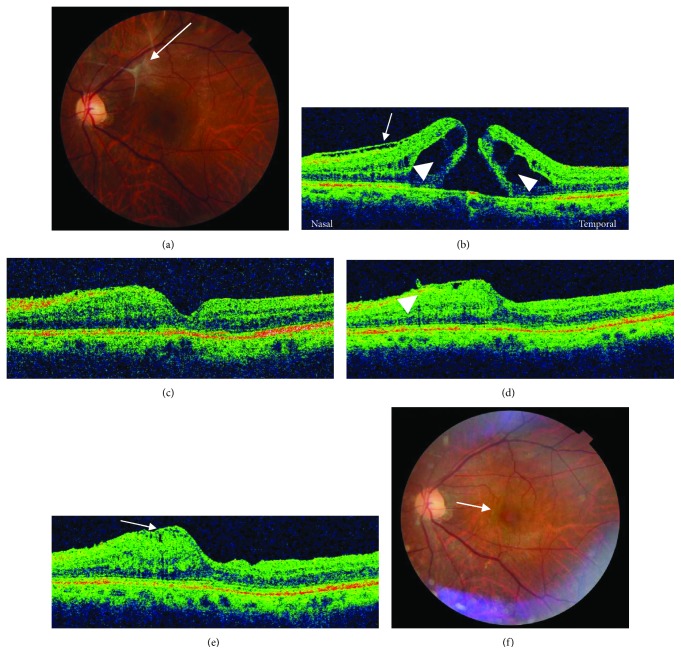
Preoperative and postoperative fundus and OCT images. A 66-year-old man with a MH associated with an ERM (arrow) in high myopia (a). The preoperative minimal and maximal MH diameters are 214 *μ*m and 1,235 *μ*m, respectively. An ERM (arrow) and cystoid retinal change (arrowheads) are seen (b). The axial length is 26.1 mm and the decimal BCVA is 0.4. At postoperative week 2, the MH is closed (c). The decimal BCVA has improved to 1.0. At postoperative week 4, retinal thickening on the inverted side of the ILM flap is seen (arrowhead) (d). At postoperative week 8, the retinal thickening has increased and the stump of shrunken inverted ILM flap was observed (arrow) (e, f). The foveal deformation stopped after postoperative week 12, and the decimal BCVA is maintained at 1.0.

During the first surgery, no complications, e.g., peripheral retinal tear and detachment, occurred in any case. Spectral-domain OCT showed postoperative restoration of the external limiting membrane (ELM) and ellipsoid zone (EZ) in 10 (50%) and 7 (35%) eyes at the final visit, respectively. The restoration of the EZ was always preceded by that of the ELM. In chronic MH cases, restoration of those two structures did not occur (Table 3). In a 66-year-old man with a MH associated with an ERM in high myopia, gradual foveal deformation occurred, i.e., elevation and displacement toward the optic disc on the nasal side of the fovea after MH closure (Figure 3). The foveal deformation stopped after postoperative week 12, and the decimal BCVA was maintained at 1.0, which negated the need for additional surgery. Except for this case, no serial complications developed.

The functional improvements are summarized in Table 2. In general, a significant postoperative improvement in the BCVA was seen in eyes with large MHs and MHs in high myopia (*p* < 0.05, Wilcoxon signed-rank test). The BCVA also improved in eyes with chronic MHs and the eye of the patient who could not maintain postoperative prone positioning, although the difference did not reach significance because of the small sample size.

## 4. Discussion

ILM peeling is the main surgical approach for treating MH. However, for large MHs, chronic MHs, MHs in high myopia, and MHs in patients unable to maintain postoperative prone positioning, the literature has reported relatively low closure rates [1–6, 9]. Michalewska et al. first reported the effectiveness of the inverted ILM flap technique for treating large idiopathic MHs and myopic MHs [10, 11]. In a comparative study of the ILM peeling technique versus the inverted ILM flap technique for treating full-thickness large MHs, the MH closure rate and VA improvement rate after the inverted ILM flap technique were significantly higher than those following ILM peeling [14]. The current findings agreed with the effectiveness of the inverted ILM flap technique for treating chronic, large, or highly myopic MHs and MHs in patients unable to maintain postoperative prone positioning. In the original inverted ILM flap technique, after circumferential ILM peeling, the trimmed ILM flaps using remnant ILM left at the MH edge were inverted and placed over the surface of the MH from all sides. A difficulty in the original technique was that the ILM flap detached in 14% (7 of 50 eyes) of cases during fluid-air exchange [10]. Recently, using the perfluoro-*n*-octane or ophthalmic viscosurgical devices effectively prevented retroversion and detachment of the inverted ILM flap until the fluid-air exchange was completed [19, 20]. However, in the current temporal inverted ILM flap technique, one large (two-disc-diameter) single-layered ILM flap with a wider connection to the retina was inverted to cover the MH and did not detach spontaneously or easily flip back with injection of the viscoelastic material during fluid-air exchange. The higher reliability and simplicity of this procedure may have contributed to the good closure rates of MHs in the current study.

The mechanism of the original method of MH closure is that the filled ILM flap in the MH space might stimulate the proliferation of glial cells that fill MHs. This packing method resulted in the multilayered membrane observed in the OCT [10] images obtained postoperatively, and the small trimmed ILM flaps that migrated to the bottom of the MH were obstacles to natural MH closure and/or functional recovery of the outer retina [18]. However, in the temporal inverted ILM flap technique, one large single-layered ILM flap provides a more regular structure for glial cell proliferation and does not reach the bottom of the MH or become an obstacle to natural MH closure. In addition, several studies have shown that complete restoration of both the ELM and EZ is prevented when the glial cell proliferation affects all retinal layers. The restoration of those structures has been reported to predict good postoperative visual outcomes; thus, the manner in which MHs close, i.e., whether glial cell proliferation affects some or all retinal layers, may be an important factor that predicts the visual prognosis, because partial proliferation would not affect the restoration of the outer retinal band [21–23].

The exact mechanism of the MH closure using the temporal inverted ILM flap technique is not precisely understood. Serial OCT observations of a current case (Figure 2) showed the course of the early structural recovery of the macula after MH surgery with the temporal inverted ILM flap technique. In the current cases, MH closure started from the proliferation of the hyperreflective tissue in the MH space just beneath the covered ILM flap (Figure 2(d)), and retinal layer remodeling then was induced gradually (Figure 2(e)). With the current technique, any glial cells adhering to the outer side of ILM flap cannot migrate to the fully sealed MH space; thus, glial cells inside the MH wall were speculated to proliferate. However, adherent cells proliferate and migrate minimally without a basement membrane, e.g., lens epithelial cells cannot migrate into an open area after posterior capsulotomy; thus, the mechanism of the temporal inverted ILM flap technique may be that a scaffold is provided as a basement membrane for MH wall gliosis without providing a possible obstacle to the bottom of the MH by inverting a single-layered ILM flap. This mechanism may explain the results of a study by Kase et al. [24], which suggested that glial cells placed on the hole may produce intermediate filaments and provoke tissue remodeling within the MH, and Shiode et al. [25] proposed that the ILM functions as a scaffold for the proliferation and migration of Muller cells when the original inverted ILM flap technique is performed. In the ILM peeling technique, in which the retina is relaxed and glial cell proliferation is induced by complete peeling, the ILM which is the most rigid part of the retina is involved in the mechanism of the MH closure [26, 27]. Complete ILM peeling relaxes the retina more than incomplete ILM removal, which occurs during the temporal inverted ILM flap technique, but a higher rate of MH closure has been reported with the inverted ILM flap technique than with the ILM peeling technique for treating chronic, large, or highly myopic MHs. This may indicate that more glial cell proliferation occurs in association with the temporal inverted ILM flap technique.

The inverted ILM flap technique may have some limitations. The rates of ELM and EZ recovery were significantly lower in the original inverted group than in the ILM peeling group [14]. Another limitation was decreased VA. Deshpande and Narayanan [15] and Hirano et al. [16] reported worsening postoperative VA compared with preoperatively. Imai and Azumi [17] observed expansion of RPE atrophy. In the current study, postoperative restoration of the ELM and EZ occurred in 10 (50%) and seven (35%) eyes at the final visit, respectively. These outcomes were similar to those of the ILM peeling technique. In addition, we did not observe VA worsening or expansion of RPE atrophy in the current cases, thus providing a single-layered flap covering the MH as a scaffold for MH wall gliosis without interposing tissue within the hole may provoke the partial proliferation caused by the manner in which the glial cells fill the hole from the retinal surface to the RPE side [22]. Achieving single-layered flap closure intraoperatively may be an important factor in the anatomic and VA improvement seen with the temporal inverted ILM flap technique. However, gradual foveal deformation after MH closure was observed in a case with high myopia associated with an ERM in the current study. ERMs contain glial cells, myofibroblasts, and hyalocytes seen histopathologically [28]. Regarding the ERM surgery, a meta-analysis reported that the rate of ERM recurrence after the initial surgery was higher in the group in which the ILM was not peeled compared with the group in which the ILM was peeled [29]. Residual ERM fragments on the ILM after ERM peeling were thought to proliferate and form the recurrent ERM [30]. The argument in favor of ILM peeling has been that the ILM may serve as a scaffold for cellular proliferation. In the current study, although the inverted ILM flap technique was performed after ERM peeling, cellular proliferation and constriction of the inverted ILM were observed only on the side in which the ILM was not peeled and where the ILM flap was inverted. This may support the theory that the ILM serves as a scaffold for ERM recurrence. In the temporal inverted ILM flap technique, the ILM is functioned by providing a single-layered flap covering the MH to serve as a scaffold for MH wall gliosis. However, in the cases of MHs associated with ERMs, the ILMs may be risk factors for the gradual foveal deformation after MH closure resulting from cellular proliferation. Recently, because the same treatment outcome was reported between the inverted ILM flap technique and ILM peeling to treat small idiopathic MHs (<400 *μ*m) and nonmyopic MHs (axial length, <26 mm), the indications for the inverted ILM flap technique are widening. However, we should note that postoperative retinal contraction might occur on the nasal side of the MH despite closure due to cell reproliferation on the unpeeled ILM when an ERM is present. This could ultimately affect both the anatomic and functional outcomes despite MH closure, inducing metamorphopsia or MH reopening. Further studies with longer follow-up are needed to test this hypothesis.

Because of the retrospective nature of the current study, the small number of cases, and the absence of a case-control design, the data require careful interpretation. We reported the efficacy of the single-layered inverted ILM flap technique for treating chronic, large, or highly myopic MHs and MHs in patients unable to maintain postoperative prone positioning and retinal contraction, which have not been reported. Further studies are needed to minimize the surgical complications and understand the mechanism of this technique.

## 5. Conclusions

The single-layered inverted ILM flap technique is a simple surgical strategy, provides a scaffold for retinal gliosis, and possibly facilitates bridge formation between the walls of the MH just beneath the ILM flap. Regarding the MH closure rate and better VA improvement, this technique should be an effective and safe method to treat chronic, large, or highly myopic MHs and MHs in patients unable to maintain postoperative prone positioning. In the MHs associated with ERMs, gradual foveal deformation developed on the side where the ILM was not peeled and the side where the ILM flap was inverted after MH closure. Further studies are needed to minimize surgical complications and understand the mechanism of this technique.

## Figures and Tables

**Table 1:** Patient baseline clinical characteristics.

Factors	All cases (*n*=20)
Mean age ± SD, years	66.8 ± 9.9
Range	48–82
Gender (male/female)	8/12
Mean BCVA ± SD, logMAR	0.73 ± 0.35
MH type no. (%)	
Large MH	6 (30%)
Chronic MH	2 (10%)
MH in high myopia	11 (55%)
MH in patient unable to maintain postoperative prone positioning	1 (5%)
Mean MH diameter (±SD)	
Large MH (*n*=6)	
Minimal diameter, *μ*m	480 ± 74
Maximal diameter, *μ*m	858 ± 324
Chronic MH (*n*=2)	
Minimal diameter, *µ*m	845 ± 43
Maximal diameter, *μ*m	1470 ± 309
MH in high myopia (*n*=11)	
Minimal diameter, *µ*m	278 ± 154
Maximal diameter, *µ*m	897 ± 740
MH in patient unable to maintain postoperative prone positioning (*n*=1)	
Minimal diameter, *µ*m	405
Maximal diameter, *µ*m	867
Lens status (phakic: IOL)	14 : 6

SD, standard deviation; IOL, intraocular lens, pseudophakia.

**Table 2:** Surgical outcomes.

Characteristic	Before surgery	Final visit	*p* ^*∗*^
MH closure (%)			
Large MH (*n*=6)		6 (100%)	
Chronic MH (*n*=2)		2 (100%)	
MH in high myopia (*n*=11)		11 (100%)	
MH in patient unable to maintain postoperative prone positioning (*n*=1)		1 (100%)	
Mean BCVA ± SD, logMAR (improved ≥0.3 logMAR, %)			
Large MH	0.59 ± 0.27	0.26 ± 0.31 (83.3%)	0.0313
Chronic MH	0.91 ± 0.55	0.55 ± 0.21 (50%)	0.2500
MH in high myopia	0.77 ± 0.38	0.35 ± 0.37 (81.8%)	0.0005
MH in patient unable to maintain postoperative prone positioning	0.82	0.52 (100%)	0.5000

^*∗*^Wilcoxon signed-rank test.

**Table 3:** Structural restoration of the bands at final visit.

Line or band no. eyes (%)	Large MH (*n*=6)	Chronic MH (*n*=2)	MH in high myopia (*n*=11)	MH in patient unable to maintain postoperative prone positioning (*n*=1)
ELM	4 (67%)	0 (0%)	5 (45%)	1 (100%)
EZ	2 (33%)	0 (0%)	4 (36%)	1 (100%)

## Data Availability

The data used to support the findings of this study are available from the corresponding author upon request.

## Supplementary Materials

Supplementary Materials This surgical video of the temporal inverted flap technique shows removal of residual vitreous cortex, creation of a semicircular ILM notch two disc diameters from the MH in a temporal area using a 25-gauge MVR knife, inversion of the ILM flap in a nasal direction to fully cover the MH, placement of a low-molecular-weight viscoelastic material on the inverted flap for stabilization, and fluid-air exchange.
